# Thermodynamic Study of Leaching Conditions of Galena with Citrate Ions and Hydrogen Peroxide as Oxidizing Agent

**DOI:** 10.3390/ma15217704

**Published:** 2022-11-02

**Authors:** O. J. Solís-Marcial, A. Nájera-Bastida, Alfonso Talavera-López, Benito Serrano Rosales, Jose A. Hernandez, R. Zarate-Gutiérrez

**Affiliations:** 1 Instituto Politécnico Nacional-UPIIZ, Ingeniería Metalúrgica, Blvd. del Bote 202, Cerro del Gato, Zacatecas 98160, Mexico; 2 Unidad Académica de Ciencias Químicas, Universidad de Zacatecas, Campus UAZ Siglo XXI, Zacatecas 98160, Mexico; 3 Instituto Politécnico Nacional-UPIIG, Guanajuato 36275, Mexico; 4 Instituto Politécnico Nacional-CeCyT-18, Blvd. del Bote 202, Cerro del Gato, Zacatecas 98160, Mexico

**Keywords:** galena, leaching, pH, citrate ion, hydrogen peroxide, activation energy

## Abstract

Galena is the most important mineral for lead production, as it is the main source of lead in the world. Currently, the concentrates of this mineral are mainly treated using pyrometallurgical methods, creating several environmental problems, such as the generation of toxic and greenhouse gases. In addition, these processes involve high energy consumption, which limits their applicability. Hydrometallurgical routes are proposed as alternative processes for obtaining some metals such as silver, copper, gold, etc. The drawback of these processes is that the minerals tend to be passive in aqueous media. To mitigate this issue, researchers have used extreme conditions of pressure and temperature (6 atm. and 155 °C) or the use of very corrosive conditions. In this sense, the use of complexing agents that dissolve the metals of interest has been proposed. Citrate ion is one of the most promising complexing agents for galena leaching, obtaining high percentages of dissolution in relatively short times. Unfortunately, there has not been enough investigation about the concentration optimization of the complexing in the pH range from 5 to 9. In this sense, thermodynamic diagrams, such as the Pourbaix diagrams, are very useful for this purpose. Therefore, in this work, the effects of pH and temperature on the leaching of galena in citrate ion solutions are studied thermodynamically and experimentally. The experimental work was carried out with pure galena samples with a particle size of +149 − 74 µm (−100 + 200 mesh). The results show that higher recoveries were obtained working at a pH of 8 and at temperatures of 30 and 40 °C. The thermodynamic and experimental data demonstrated that the existence of an optimal concentration of citrate ion, due the extraction of lead from galena, has a greater reaction rate at a relatively low initial concentration of 0.3 M. This is due the formation of the complex lead citrate 1 ($\mathrm{Pb}{\left(\mathrm{cit}\right)}^{-}$).

## 1. Introduction

Lead is one of the most important metals; its main application consists in the manufacture of batteries for automotive and other electronic devices. Galena (PbS) is the most common lead ore in nature and can be frequently found in various mineral deposits [1,2]. Currently, one treatment of the lead ores involves some unit operations, such as the froth flotation process, in which a galena concentrate is obtained. Subsequently, this concentrate is subjected to pyrometallurgical treatments to obtain metallic lead. However, these processes have several disadvantages, such as a high energy requirement to obtain bullion lead, which, in order to increase its purity, requires refining. In addition, some environmental problems are generated, such as the production of ${\mathrm{SO}}_{2}$ and volatile lead. To prevent these compounds from polluting the environment, it is necessary to implement countercurrent washing towers, which has an impact on operating costs.

Hydrometallurgical methods at low temperature are considered an alternative to pyrometallurgical processes for obtaining lead, because they require less energy and do not generate toxic gases. In addition, these processes have a higher selectivity to obtain the metals. Unfortunately, at present, the schemes proposed to produce lead by hydrometallurgy lack a projection for industrial levels, because temperatures close to the boiling point of water, high concentrations of strong acids and/or chloride ions are required to obtain high extraction percentages [3,4,5,6,7,8,9,10,11]. Sulfate, nitrate and chloride ions have been proposed for the leaching of galena [12,13,14,15,16,17,18,19]. However, most of them hardly dissolve lead. Other investigators have proposed the use of carboxylic and organic acids such as oxalic, citric, ascorbic, acetic, fumaric and tartaric acids [12]; one of the most widely used is acetic acid. Evans and Masters leached finely crushed galena by converting it to soluble lead acetate and the sulfur ion to its elemental form. Another enhancement of the leaching of galena with acetate ions is the addition of an oxidizing agent. Aydogan et al. performed acetate ion leaching tests using H_2_O_2_ as the oxidizing agent, finding that peroxide can oxidize sulfur to sulfate, which can limit the leaching. Ferric metasulfonate has also been proposed to leach galena, achieving nearly 90% extraction in the first 10 min of experimentation, with an apparent activation energy of 36.15 kJ/mol [20,21,22,23]. A problem with this process is the production of sulfate ions, which can limit the leaching kinetics. On the other hand, citrate ion in conjunction with hydrogen peroxide, as an oxidizing agent, have demonstrated a great functionality for the leaching of lead compounds, such as galena. Zarate and Lapidus, found that the mixture of citrate ion and hydrogen peroxide allows selective leaching, obtaining a lead extraction percentage of 100% in three hours. These researchers worked mainly in acidic pH ranges, and they did not consider the possible effects of basic pH nor the variation in the citrate ion concentration. Therefore, the objective of the present work is to study, thermodynamically and experimentally, the effect of the concentration of the complexing and leaching agent, such as sodium citrate, and temperature on the leaching of pure galena.

## 2. Experimental Method

**Figure 1:**
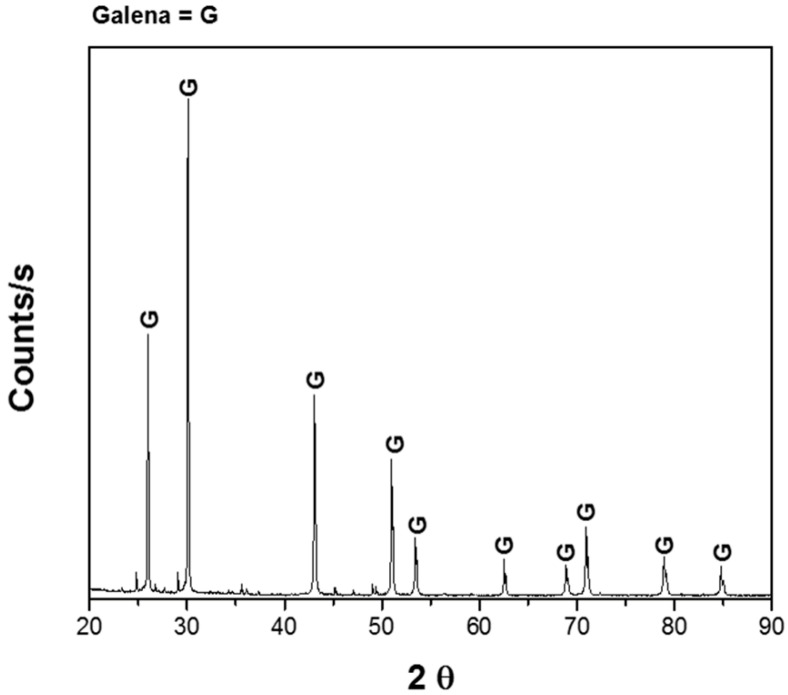
X-ray diffractogram of galena.

Experiments were performed with 1 g of pure galena provided by Wards Scientific Inc. (Rochester, NY, USA); it was crushed to a particle size of −149/+74 μm. The mineralogical analysis was carried out using X-Ray Diffraction (XRD), supporting ore purity (Figure 1) and Atomic Absorption Spectrophotometry (AAS, Buck Scientific 219), obtaining the following mineral composition; 91.5% galena and 8.5% impurities. The dissolution experiments were carried out with a solution volume of 0.1 L with different concentrations of sodium citrate at different pH and constant agitation of 600 rpm. In order to adjust the pH, dilute sulfuric acid and sodium hydroxide solutions were used, as appropriate. Deionized water and reagent grade chemicals were used to prepare all the solutions. Samples of solution were withdrawn from the reactor at different times up to 180 min, and lead was analyzed with AAS; this was carried out in triplicate, and the average is shown in the results. The ranges of each variable were: pH of the solution 5–9, temperature 20–40 °C, concentration of sodium citrate 0 to 1 M. 

## 3. Results and Discussions

### 3.1. Thermodynamic Study

When the objective of a process is to dissolve a mineral or metal, it is important to understand the effect of oxidation-reduction potential (ORP), and the pH of the solution, as both potential and pH directly impact the dissolution of the mineral of interest. In addition, it must be considered that in certain aqueous media, the mineral may be passivated rather than dissolved. In order to achieve this goal, Pourbaix diagrams are useful tools which relate oxidation-reduction potential and pH. It can predict which metal phases will form from a thermodynamic point of view, under certain conditions [23]. 

**Figure 2:**
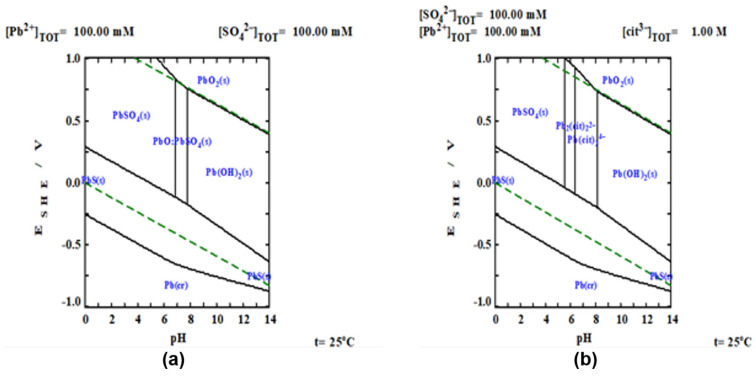
E_SHE_-pH predominance diagrams. (**a**) $\mathrm{Pb}/H_{2}O/{\mathrm{SO}}_{4}$ system and (**b**) $\mathrm{Pb}/H_{2}O/{\mathrm{SO}}_{4}/{\mathrm{cit}}^{3-}$ system. Where: ${\left[{\mathrm{Pb}}^{2+}\right]}_{\mathrm{TOT}}=\mathrm{Total}\;\mathrm{Lead}\;\mathrm{Concentration}$; ${\left[{\mathrm{SO}}_{4}^{2-}\right]}_{\mathrm{TOT}}=\mathrm{Total}\;\mathrm{Ion}\;\mathrm{Sulfate}\;\mathrm{Concentration}\;$; ${\left[{\mathrm{Cit}}^{3-}\right]}_{\mathrm{TOT}}=\mathrm{Total}\;\mathrm{Ion}\;\mathrm{Citrate}$.

To carry out the thermodynamic study of the experimental results, Pourbaix diagrams were constructed using the MEDUSA software [24]. The algorithm that uses the software is the reported by Eriksson, where Gibb’s Free Energy is minimized and all possible equilibria between the components are considered for the aqueous systems, considering or not the use of the complexing agent, as shown in Figure 2a,b. For the $\mathrm{Pb}/H_{2}O/{\mathrm{SO}}_{4}$ system, Figure 2a, the formation of sulfates, oxides and hydroxides of lead as ${\mathrm{PbSO}}_{4}$, PbS, ${\mathrm{PbO}}_{2}$, $\mathrm{Pb}{\left(\mathrm{OH}\right)}_{2}$, is observed. At acidic pH, the formation of lead sulfates and sulfides is favored and at basic pH, lead hydroxides and oxides are formed, thus limiting the dissolution of lead. On the other hand, with the presence of a complexing agent, such as citrate ion, (Figure 2b), it is observed that thermodynamically, there are wider intervals where lead is found as a complexed ionic phase (${\mathrm{Pb}}_{2}{\left(\mathrm{cit}\right)}_{2}^{2-},\;\mathrm{Pb}{\left(\mathrm{cit}\right)}_{2}^{4-}$), demonstrating the beneficial effect of the use of complexing agents in the dissolution of galena.

### 3.2. Leaching Results

Based on the Pourbaix diagram for the $\mathrm{Pb}/H_{2}O/{\mathrm{SO}}_{4}/{\mathrm{Cit}}^{3-}$ system (Figure 2b), it can be determined that the lead citrate complex is favored in the pH range from 5 to 9 and has a potential higher than 0.1 V.

**Figure 3:**
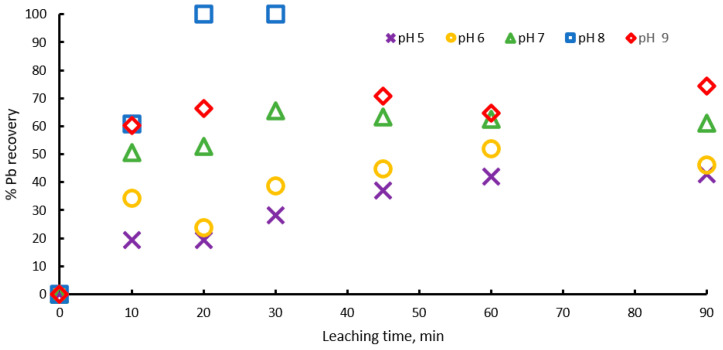
Effect of pH on lead dissolution from pure galena at 20 ° C. Experimental conditions: particle size: −149/+74.1 g of mineral, 0.1 L of solution, 1.0 M $Na_{3}Cit$, 0.6 M $H_{2}O_{2}$.

To validate the above, dissolution experiments were carried out in the pH range from 5 to 9, with 1 M sodium citrate, 0.6 M $H_{2}O_{2}$, and at 20 °C. Figure 3 shows the percentage of lead extraction increasing with time, with pH values of 5, 6, 7 and 8, and lead extraction percentages of 40, 45, 60 and 100 percent being obtained, respectively, which indicates that extraction percentage increases with pH until pH = 8, but at pH = 9 the process Is inhibited and an extraction of 75% is achieved, probably because at pH above 9, lead hydroxides begin to form, precipitating or forming a passivation layer on the surface of the ore. These results suggest that there is an optimum pH = 8 for galena leaching, due the formation of the ${\mathrm{PbCit}}^{-}$ complex being favored.

**Figure 4:**
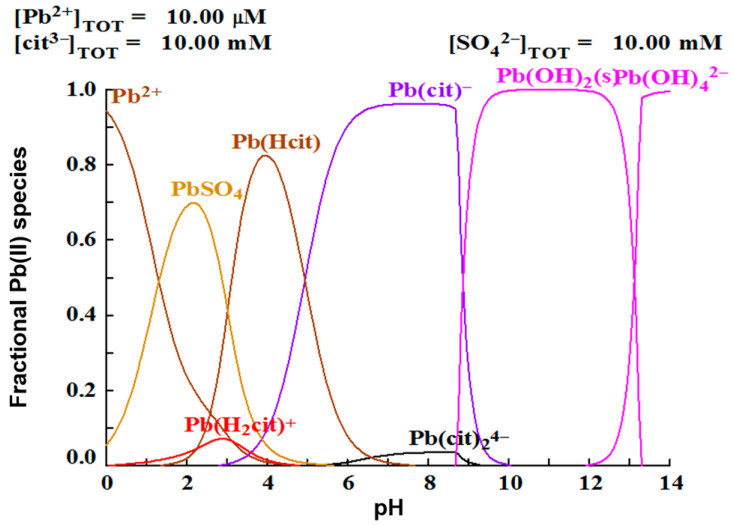
Species distribution diagram of lead in presence of citrate and sulfate ions.

A justification can be made analyzing the speciation diagram in Figure 4. It is observed that the $\mathrm{Pb}{\left(\mathrm{cit}\right)}^{-}$ complex prevails over all other lead species at pH 8, while at pH 9, $\mathrm{Pb}{\left(\mathrm{OH}\right)}_{2}$ formation is favored. Hsieh and Huang found that the dissolution of galena is favored at acidic pH, without the presence of a complexing agent or oxidizing agent. The best lead dissolution results were obtained at a pH of 2.5, due the formation of PbSO_4_, which could represent considerably corrosive conditions. On the other hand, it has been found that lead can be dissolved at neutral pH with the addition of a complexing agent, such as citrate ion [25,26,27]. These researchers explain this phenomenon, with a thermodynamic analysis with the use of speciation diagrams, establishing that in a pH range of 6.5–8.5 the species ${\mathrm{Pb}}_{2}{\left(C_{6}H_{5}O_{7}\right)}_{2}^{2-},\;\mathrm{Pb}{\left(C_{6}H_{5}O_{7}\right)}^{-},\;\mathrm{Pb}{\left(C_{6}H_{5}O_{7}\right)}_{2}^{4-}$ are formed, which facilitates the dissolution of lead, in agreement with the results of this work. 

### 3.3. Effect of Complexing Ion Concentration

**Figure 5:**
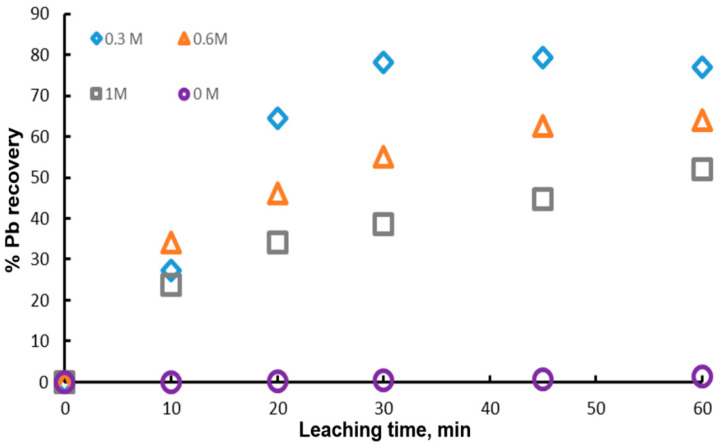
Effect of the initial concentrations of citrate ion on lead dissolution from pure galena at 20 °C. Experimental conditions: particle size: −149/+74.1 g of mineral, 0.1 L of solution, 0.3–1.0 M $Na_{3}Cit$, 0.6 M $H_{2}O_{2}$.

One of the most important variables in the dissolution of galena is the concentration of the complexing ion $C_{6}H_{5}O_{7}^{3-}$, since it can modify the degree of leaching and thus the cost of the process. Figure 5 shows the experimental results obtained with different initial concentrations of citrate ion (0 M, 0.3 M, 0.6 M and 1 M), 1 g of mineral, 0.1 L of leaching solution, 0.6 M of $H_{2}O_{2}$ and a temperature of 20 °C. It is possible to appreciate that the highest value of the percentage of Pb recovery is reached with 0.3 M and it decreases with the citrate ion concentration. This indicates that the presence of citrate ion above 0.3 M is detrimental to the dissolution of galena. However, for the experiment with 0 M citrate ion, the lead extraction was null, demonstrating that the addition of citrate ion to the leaching solution is necessary until 0.3 M. These results suggest that there is an optimum concentration of the complexing agent for the dissolution of galena. In this context, Zarate and Lapidus, found that the highest anglesite dissolution was obtained at pH 7 and 1 M sodium citrate. On the other hand, Kourgiantakis et al. observed that there is an optimum stoichiometric ratio during the synthesis of lead citrate crystals, whereby the production of crystals is higher. Constance et. al., found that there is a higher amount of lead extracted using lower concentrations of citrate ion, obtaining extraction percentages of 90 and 100% with 0.5 and 0.25 M, respectively. 

**Figure 6:**
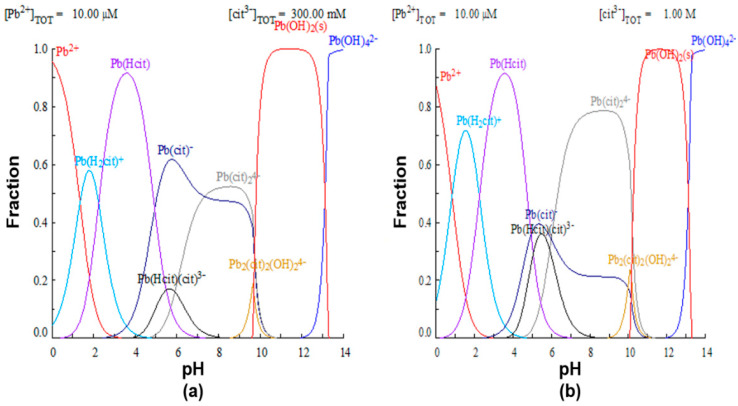
Species distribution diagram of lead with different concentrations of citrate ion. (**a**) 0.3 M and (**b**) 1.0 M.

To justify the proposed premise that the efficiency of lead leaching depends on the formation of the $\mathrm{Pb}{\left(\mathrm{cit}\right)}^{-}$ complex, lead speciation diagrams were constructed using the MEDUSA software, with 0.3 and 1M citrate ion initial concentrations, shown in Figure 6. In the first case, it is observed that, for a 0.3 M concentration of complexant (Figure 6a), at a pH of 8, the formation of the $\mathrm{Pb}{\left(\mathrm{cit}\right)}^{-}$ species is favored, while at a higher concentration of the complexing agent (Figure 6b), the formation of the $\mathrm{Pb}{\left(\mathrm{cit}\right)}_{2}^{4-}$ and $\mathrm{Pb}{\left(\mathrm{cit}\right)}_{2}{\left(\mathrm{OH}\right)}_{2}^{4-}$ complexes occurs. 

### 3.4. Temperature Effect

**Figure 7:**
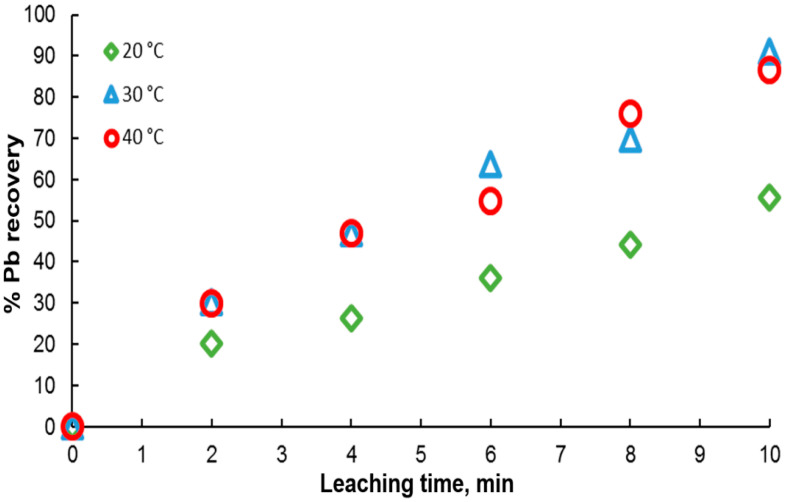
Effect of temperature on lead dissolution from pure galena. Experimental conditions: particle size: −149/+74.1 g of mineral, 0.1 L of solution, 0.3 M $Na_{3}Cit$, 0.6 M $H_{2}O_{2}$.

Ore leaching is very sensitive to temperature. Therefore, to determine the effect of this variable on galena dissolution, experiments were carried out in the range of 20 to 40 °C in solutions containing 0.3 M citrate ion and 0.6 M $H_{2}O_{2}$. As shown in Figure 7, the results indicate an increase in the percentage of lead extraction with time, in agreement with an endothermic behavior, where galena dissolution is favored with temperature. It is found that at 20 °C, the lowest dissolution rate is obtained, reaching a maximum of 50% at 10 min. For the other two temperatures, a very similar leaching kinetics is observed between them, with very little variation in the extraction percentages, reaching around 90% extraction at 10 min. 

### 3.5. ORP Monitoring

**Figure 8:**
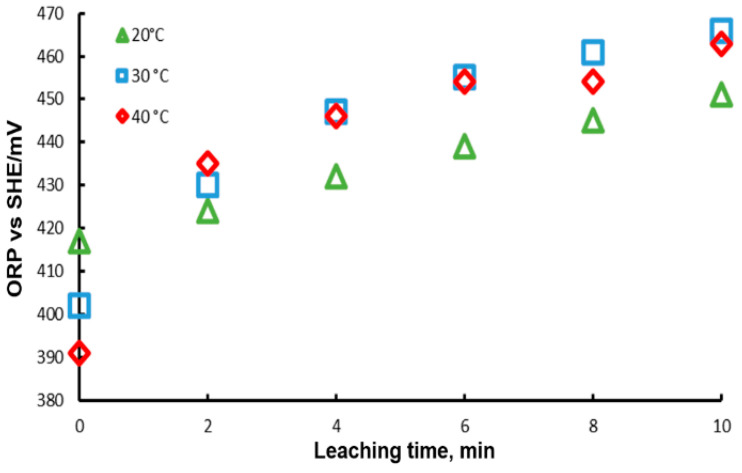
ORP variation during lead dissolution from pure galena at different temperatures. Experimental conditions: particle size: −149/+74.1 g of mineral, 0.1 L of solution, 0.3 M $Na_{3}Cit$, 0.6 M $H_{2}O_{2}$.

The Oxidation-Reduction Potential (ORP) of the solution was monitored during these experiments; the results are presented in Figure 8. In this Figure, it is observed that at the beginning of the experiment, the ORP decreases with temperature, reaching a higher potential with a lower temperature. This can be explained by the Nerst equation, which predicts that ORP decreases with temperature. On the other hand, it is observed that ORP increases with time, which can be associated with the concentration of the ${\mathrm{Pb}}^{2+}$ ion in each of the cases. For the case of 20 °C, the lowest ORP measurements were obtained, while for 30 and 40 °C the values are very close between them, in agreement with the leaching results. This behavior may be due to the fact that the concentrations of lead ions modify the potential, according to the Nerst equation which points out that potential is dependent on ion concentration. 

### 3.6. Kinetic Analysis

Galena dissolution in citrate solutions is a heterogeneous reaction and the important kinetic aspects can be observed using the shrinking core model. According to this model, the dissolution processes are controlled by diffusion across the solution boundary, diffusion through the ash layer or chemical reaction at the surface [27]. Other variables included in the model are particle geometry, porosity and permeability of the mineral [28,29,30,31,32]. In this sense, the shrinking core models were adjusted; when diffusion and reaction controlled the process, with different geometrical shape, this impacted on the kinetic analysis, with the shrinking core model being controlled by reaction with cylindrical particle geometry; this is represented by Equation (1).

(1) $$1-{\left(1-X_{B}\right)}^{\frac{1}{2}}=\frac{b\cdot k^{\prime \prime }\cdot C_{A}\cdot t}{R\cdot \rho_{B}}$$

where *X_B_* is the fraction of reacted galena, t is the time (min), *C_A_* is the average (apparent) concentration of the reagent (mol m^−3^), *R* is the radius of the solid particle (m), *b* is the stoichiometric coefficient of the solid reacting with an amount of fluid reagent, *k*″ is the kinetic rate, and *ρ*_B_ is the molar density of PbS. 

**Figure 9:**
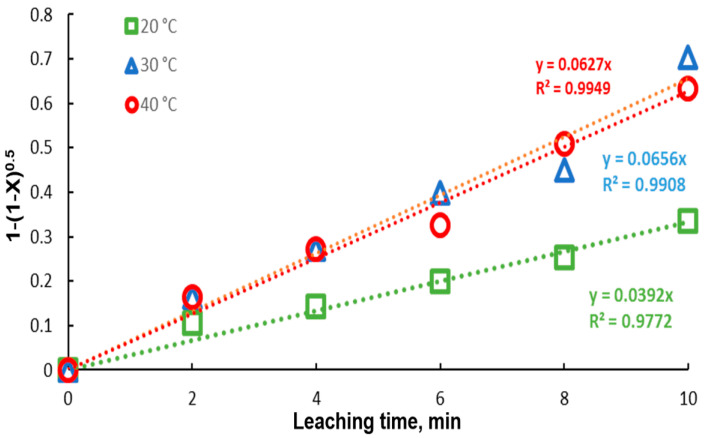
Fitting of the core shrinking model to data for different temperatures (20–40 °C). Experimental conditions: particle size: −149/+74.1 g of mineral, 0.1 L of solution, 0.3 M $Na_{3}Cit$, 0.6 M $H_{2}O_{2}$.

In Figure 9, the model represented by Equation (1) was fitted to the lead recovery data at the different experimental temperatures. An acceptable fit of the model is presented, with correlation coefficients very close to 1. It is observed that, for the temperatures of 30 and 40 °C, the kinetics are similar, obtaining kinetic constants close to each other. Due to the great similarity between the extraction percentages at 30 and 40 °C, the kinetic constants are very close in magnitude, 0.0656 and 0.0627, respectively. Using the Van’t Hoff equation (Equation (2)), the activation energy was determined, with a value of $-3565.105\frac{J}{mol\;K}$.

(2) $$ln\frac{k_{1}}{k_{2}}=\frac{E}{R}\left(\frac{1}{T_{2}}-\frac{1}{T_{1}}\right)$$

(3) $$E=ln\frac{k_{1}}{k_{2}}\left(\frac{R}{\frac{1}{T_{2}}-\frac{1}{T_{1}}}\right)$$

$$E=ln\frac{0.0656}{0.0627}\left(\frac{8.314\frac{J}{mol\;K}}{\frac{1}{313\;K}-\frac{1}{303\;K}}\right)=-3565.105\frac{J}{mol\;K}$$

where E is the activation energy, R is the ideal gas constant, $k_{1}$ and $k_{2}$ are the kinetic constant at Temperature 1 $\left(T_{1}\right)$ and Temperature 2 $\left(T_{2}\right)$, respectively.

An interpretation of negative activation energy is realized by Tolman, where it is proposed as the average energy of reactants and the average energy of the transition state. Thus, the activation energy will be negative if reactants with low energy react faster than those with high energy [30,33]. The negative activation energy involves two or more elementary reactions, where the negative value is due at the addition of each activation energy corresponding to elementary reaction. According to Benson and Devis, this phenomenon involves two or more steps in the transition state, where the first step is actually the formation of a weakly bound complex; in the next step, the transition state has a barrier below the first transition state, leading to the negative activation energy.

Trent and Abraham evaluated a monolith-supported Pt/Al_2_O_3_ for wet oxidation of cellulose. They found that the rate constant deceased with temperature and calculated a negative value of activation energy for the catalytic decomposition; this result is possible when the overall reaction is comprised of several kinetically significant reaction steps. To justify this result, they propose a more detailed reaction pathway scheme, where cellulose decomposition occurs through acid catalyzed depolymerization, forming soluble organic species in water, and this leads to the inverse behavior of temperature, and eventually negative activation energy of cellulose conversion.

This agrees with the formation of the lead citrate complex, $Pb{\left(\mathrm{cit}\right)}^{-}$; in the leaching of galena with sodium citrate and justifies the negative value of the activation energy. Actually, the activation energy in mineral leaching is normally considered positive. In this sense, galena leaching is achieved by the use of oxygen peroxide and it is monitored by the formation of hydroxyl radicals. The reactions with this radical present negative activation energy.

## 4. Conclusions

The dissolution of pure galena with hydrogen peroxide and citrate ions was studied. Based on the results and discussion, the following conclusions are derived.

Thermodynamic analyses are of great importance, since they allow predicting the phases formed as a function of ligand concentration and pH. It was found that there is an optimum concentration of this ligand, which is 0.3 M and a pH of 8, because at this concentration of ion citrate and pH = 8, the formation of the $Pb{\left(cit\right)}^{-}$ complex is favored, obtaining an almost complete dissolution (95%) of lead in short times of 10 min under these conditions. Thus, it was possible to establish the optimum conditions for galena leaching. The kinetic study presented a negative activation energy of $-3565.105\frac{J}{mol\;K}$, in the temperature range of 30–40 °C, suggesting that leaching occurs in two or more steps.

## Data Availability

Not applicable.
